# Parent participation plays an important part in promoting physical activity

**DOI:** 10.3402/qhw.v10.27397

**Published:** 2015-08-14

**Authors:** Anna-Karin Lindqvist, Catrine Kostenius, Gunvor Gard, Stina Rutberg

**Affiliations:** Department of Health Sciences, Luleå University of Technology, Luleå, Sweden

**Keywords:** Adolescents, empowerment, interviews, content analysis, school

## Abstract

Although physical activity (PA) is an important and modifiable determinant of health, in Sweden only 15% of boys and 10% of girls aged 15 years old achieve the recommended levels of PA 7 days per week. Adolescents’ PA levels are associated with social influence exerted by parents, friends, and teachers. The purpose of this study was to describe parents’ experiences of being a part of their adolescents’ empowerment-inspired PA intervention. A qualitative interview study was performed at a school in the northern part of Sweden. A total of 10 parents were interviewed, and the collected data were analyzed with qualitative content analysis. Three subthemes were combined into one main theme, demonstrating that parents are one important part of a successful PA intervention. The life of an adolescent has many options and demands that make it difficult to prioritize PA. Although parents felt that they were important in supporting their adolescent, a successful PA intervention must have multiple components. Moreover, the parents noted that the intervention had a positive effect upon not only their adolescents’, but also their own PA. Interventions aimed at promoting PA among adolescents should include measures to stimulate parent participation, have an empowerment approach, and preferably be school-based.

Although physical activity (PA) is an important and modifiable determinant of health (Leech, McNaughton, & Timperio, 2014), only 15% of boys and 10% of girls at age 15 in Sweden achieve the recommended levels of PA 7 days per week (Folkhälsomyndigheten, 2014). Moreover, the fact that PA is associated with a substantial number of health and academic benefits (Basch, 2011; Janssen & LeBlanc, 2010; World Health Organization, 2010) raises controversy associated with the view that schools actually support a sedentary lifestyle (Donnelly & Lambourne, 2011). Integration of PA interventions in schools can promote both health and learning, and Ickovics et al. (2014) suggested that schools and families should work together to ensure that students adopt health-promoting behaviors to achieve higher academic achievements. Schools currently prioritize academic achievements, and health is often perceived as a secondary priority at best (Basch, 2011). However, children spend approximately half of their waking hours in school, which provides an opportunity to promote PA for all children regardless of their life circumstances (Naylor & McKay, 2009). Furthermore, most schools are able to offer the equipment, facilities, and staffing needed to effectively promote PA (Carson, Castelli, Beighle, & Erwin, 2014).

Adolescents’ PA levels are associated with social influence exerted by parents, friends, and teachers (Beets, Cardinal, & Alderman, 2010). Parents are in a unique position because adolescents’ health behaviors are largely influenced by home-related factors, such as eating patterns at home, PA, and sedentary behaviors (Patino-Fernandez, Hernandez, Villa, & Delamater, 2013). Moreover, parent involvement is often recommended as a part of school-based PA interventions (Birch & Ventura, 2009). Parents’ impact on their adolescents’ PA can consist of providing different types of social support, for example encouragement and practicing together (Beets et al., 2010). Another need children expressed was for transportation by parents to sporting facilities and other arenas to enable their engagement in physical activities (Wright, Wilson, Griffin, & Evans, 2010). According to Bandura (2004), support from parents can reduce the perceived obstacles, increasing the likelihood of PA. Moreover, physically active parents can also act as positive role models; observing the behavior and learning from socially important persons might influence the PA of adolescents (Beets et al., 2010). A review by Edwardson and Gorely (2010) observed that physically active parents were more likely to have physically active children. Another review concluded that some effects of parental involvement were found in children's eating and PA behaviors but further studies on school-based interventions with parental components are needed (Van Lippevelde et al., 2012). Although research has shown that parents have an important part in their adolescents’ PA, we were unable to identify any study addressing the perspective of parents’ experiences of participation in their adolescents’ PA interventions.

## Situating this research study

We previously conducted a study where the aim was to explore the possibility of conducting an empowerment-inspired intervention and to examine the impact of the intervention in promoting PA among adolescents (Lindqvist, Mikaelsson, Westerberg, Gard, & Kostenius, 2014). The intervention was school-based and consisted of three components: contracts, encouraging peer-peer text messages, and a parental brochure. The contents of these components were created by the adolescents with support from the researchers and the teachers, using an empowerment-inspired approach. Furthermore, the development of the intervention was guided by Bandura's social cognitive theory (Bandura, 2004), which is one of the most frequently used health behavior theories. The adolescents were divided into pairs by the teachers and were asked to make a mutual written contract. The contracts included a goal for PA and a promise to support each other's PA by sending one text message to each other once each day for 1 month, to encourage PA during school hours and during leisure time. The parental brochure contained several headlines, for example: “Why is it good to be physically active?” “The relationship between PA and school performance,” and “How can parents support PA?” The brochure was sent home to the parents; however, the parents had no obligation to be active in the intervention any further. Subjective and objective PA data was collected before and after the intervention. The participants in the intervention group increased their PA compared to the control group, and the study showed that it is possible to develop and conduct an empowerment-inspired intervention to promote adolescent PA. The data collection, the content of the intervention, and the results are reported in detail elsewhere (Lindqvist, Mikaelsson, et al., 2014). As parents are known to influence their adolescents’ PA, it is valuable to explore their experiences of being a part of a school-based intervention aimed at promoting PA among adolescents.

## Aim

The aim of this study was to describe parents’ experiences of being a part of their adolescents’ empowerment-inspired PA interventions.

## Method

### Methodological framework

This study was the last part in a set of four studies with the overall aim of exploring the development of a health-promoting intervention that uses empowerment and information and communication technology, to examine the impact of the intervention, and to describe adolescents’ and parents’ experiences of the intervention. These studies applied both qualitative and quantitative methods and, according to Mengshoel (2012), mixed methods research involves the combination of qualitative and quantitative research in a single study or set of studies. The use of mixed methods research is advocated in physiotherapy, with both quantitative measurements of physical functioning and interviews about individuals’ personal experiences (Mengshoel, 2012; Rauscher & Greenfield, 2009). In this study, interviews were carried out in accordance with Kvale and Brinkmann (2009).

### Participants

This study was part of a school development and research project in one municipality of approximately 17,000 inhabitants in the northern part of Sweden. All of the staff members at the municipality's secondary school were informed about the forthcoming studies by two of the authors, and two seventh grade teachers were invited to participate as coordinators. In the first study of the project, 28 students from the two classes (13 boys and 15 girls), aged 13 and attending the seventh grade, participated in focus groups (Lindqvist, Kostenius, & Gard, 2012). The ideas of the students themselves were used to create an intervention. When the students began ninth grade, 27 students (14 boys and 13 girls) participated in the intervention group in a second study. The goals of this study were to explore the possibility of conducting an empowerment-inspired intervention and to examine the impact of the intervention in promoting PA among adolescents. After completion of the second study, all parents of the students in the intervention group were invited to participate in a qualitative study; 10 parents (four fathers and six mothers) agreed to be interviewed. These were the parents of six boys and four girls with varying PA levels. Three parents described their adolescents as being very active, two said that their adolescents were inactive, and the rest said that their adolescents were somewhere in-between. The 10 participating parents were between 40 and 55 years of age and had education levels varying from high school to higher academic education. The parents had diverse PA levels: two parents described themselves as being very active, three said that they were inactive, and the rest said that they were somewhere in-between.

### Procedure

We followed the WMA Declaration of Helsinki—Ethical Principles for Medical Research Involving Human Subjects concerning informed consent. The research ethics committee in Umeå, Sweden, approved the study before the start of the research project (date of issue: February 11, 2011; application registration number: dnr 2010-337-31Ö). Data was collected using individual interviews. We constructed a guide with questions concerning the intervention, in which the first question was “Let's pretend I know nothing. Could you please tell me about the intervention?” Examples of other questions were “What expectations did you have before?” and “In what way have you supported your adolescents’ PA?” In accordance with the recommendations of Kvale and Brinkmann (2009), follow-up questions (e.g., “Could you tell me more?” “How did you experience that?”) were posed to obtain richer material. The interviews were carried out in a room without distractions at the location the parent preferred: at the children's school, at the parents’ worksite, or at home. All interviews were conducted within 2 months of the conclusion of the intervention study. Author AKL, who had no professional connection with the students, performed the interviews, which lasted between 30 and 60 min. The interviews were sound-recorded and transcribed verbatim.

### Data analysis

The transcribed data material from the interviews was analyzed as a whole. An inductive qualitative approach was used (Elo & Kyngäs, 2008). The qualitative latent content analysis was performed according to Graneheim and Lundman (2004), and all authors took an active part in the following procedure: (1) The written material was first read through several times to obtain a sense of the overall data; (2) The text was divided into meaning units and condensed; (3) In the abstraction process, the condensed meaning units were coded and the codes were compared, contrasted, and sorted into preliminary categories. During this process, the authors strove to be close to the text; (4) By going back and forth among the preliminary categories, the codes and the text categories were identified; (5) The underlying meaning of the categories was interpreted and formulated into three subthemes, which in turn formed one main theme.

## Results

The results were formulated into one main theme and three subthemes and representative quotations from the transcribed text were used. The quotes are labeled with the number of the participant (1–10) and marked with F for female or M for male.

### Parents are one important part of a successful PA intervention

The three subthemes were combined in one main theme that shows parents having an important part to play in order for the intervention to be successful in increasing the PA of their adolescents. Adolescents have many options and demands in life that make it difficult to prioritize PA even if they know they should be physically active. Although parents stated that they felt they were important in supporting their adolescents’ PA level in general, our results show that a successful PA intervention should consist of multiple components. Furthermore, it was the parents’ experience that the intervention had a positive effect upon both their adolescents’ and their own PA.

#### PA is not always a priority

The parents recognized the advantages PA brought to their adolescents. They noted as well the obstacles faced by the children, such as lack of time and the need to prioritize PA ahead of other appealing tasks; they noted that their adolescents do not have an easy choice to make. The parents described being interested in their adolescents’ PA and recognizing several benefits of PA, such as longer and healthier life, better stamina, strength, and fitness. The parents had also noted disadvantages for their adolescents related to physical inactivity, such as being overweight, getting headaches, and being irritable. Parents feared that their adolescents would not have the physical capacity to master a physically demanding job in the future. The parents perceived that their adolescents needed support concerning their PA and emphasized the importance of creating a healthy lifestyle at a young age, because it is difficult to change behavior later in life.

**M3:** They are mostly inactive. I told my kid, you will have problems later on being so sedentary now. And the habits you create at a young age will stick, it is hard to change a habit.

Parents who had athletically active adolescents stated that they were not so worried for their own adolescents’ PA but were concerned about the young generation overall. Even some parents of adolescents who were very active in sports noted that their adolescents had a problem with physical inactivity, for example, on days when they did not have a training session or were off-season for their sport. The parents stated that they were more physically active during their own childhood relative to their children; they recognized barriers to their children's PA due to several changes that have occurred within society since their childhood. Examples that were mentioned included technology such as television, computers, and smart phones; lack of spontaneous physical activities, such as playing hockey in the street or going fishing; and less active transportation to school and other places.

**M7:** They have lost spontaneous sport. It does not exist today. There are many other things that attract; computers and mobile phones take up a lot of time. TV, for that matter: when I was growing up we had one channel, and now …. They have no easy choices to make.

Other factors that negatively influence PA include a strong commitment and heavy workload at school; some parents also noted that the girls prioritized spending time with their boyfriends over being physically active. The parents identified factors such as tight time schedules and difficulties in solving the “puzzle of life in the family” as barriers for their adolescents’ PA. Some also mentioned that lack of money and a tight budget might be a problem. The parents stated that adolescents do not always have the ability to know what is best for them and do not have the capacity to understand the long-term consequences of their actions. They believed that this could also contribute to why adults are needed to support adolescents in PA.

#### I am important, but only one piece of the puzzle

The parents were very positive about the intervention, were pleased to be included, and stated that they were mainly responsible for their adolescents’ PA in general. Simultaneously, they expressed their awareness that they were only one part of a successful PA intervention. In addition to their own support, they mentioned school and sports clubs as other important authorities. The parents felt that school was the right context for a PA intervention, because it reached every adolescent regardless of their other life circumstances, and they stated that the adolescents might listen more to an “outsider.”

**F10:** Oh, how perfect, I thought when I heard about this. A health project at school, someone from the outside saying the same thing as I am. Then maybe he will be more physically active. The teachers are annoying, parents are annoying and nagging. It is simply a new voice.

The parents thought that they were important in encouraging their adolescents’ PA. They noted that their own PA behavior had an impact on their adolescents and they wanted to act as positive role models. Furthermore, they felt that it was more effective to act as a positive role model than to attempt to convince an adolescent with facts or to nag them into being physically active. Another part of being a supportive parent was to help with practical things, such as driving the adolescents to practices and games, assisting with funds for training equipment, and arranging for adequate food for active children. The parents’ responsibility also included setting limits in areas such as time spent at the computer. When reading the parental information brochures, parents were impressed by their adolescents' knowledge and their ability to find the right details to include in the brochures. They also felt empowered, because the intervention provided several opportunities to communicate about PA with their adolescents, as well as suggestions for what they as parents could do to support their adolescents’ PA. Some of the parents also experienced a feeling of self-efficacy and a confirmation that their previous attitude and actions had been correct.

**F1:** When I read this [the parental information brochure], I thought, how funny, we have done this, intuitively we have done the right thing. It was like a confirmation; instead of feeling old-fashioned, I felt up-to-date.

One successful factor mentioned by the parents was that the intervention was built on their adolescents’ own ideas and their own choice of PA. The parents mentioned that it was an advantage that the adolescents were part of the project from start to finish, for example, that they were presented with the final results of the studies. They felt that listening and giving the adolescents responsibility was the correct approach to addressing adolescents and PA.

**
F6:** This is great. And above all that you have listened to them, taken advantage of their expertise, given power to them. Cooperation and involvement makes it fun.

Another contributing factor, according to the parents, was that the intervention included everyone instead of targeting only the least physically active children. The involvement of the majority became a unifying factor in the class and PA became a topic of conversation. The parents perceived that “fun” was an important factor in motivating adolescents to be physically active. It was important that the adolescents found an activity they perceived to be enjoyable. Some parents also stated that the young generation is driven too much by enjoyment, which might become a threat to their health. The parents perceived that measuring PA had contributed to their adolescents’ motivation to be physically active. According to the parents, a focus on competitions and challenges might be a way of further enhancing the effect of a PA intervention for adolescents. Regarding rewards, experiences differed among the parents. Although some parents had used rewards with success, others were negative towards rewarding their adolescents when they were physically active. According to some parents, rewards might be effective in the early stages of behavioral change. The parents feared that using rewards would lead to inflation (i.e., the prize for a desired action could get higher and higher), and felt that one should focus on the inner feeling of satisfaction gained from PA. Parents suggested introducing the PA intervention at an earlier stage and also implementing long-term follow-ups as a part of a successful PA intervention.

#### The intervention had a positive impact on my child and on me

The third subtheme showed that, according to the parents, the intervention had a positive effect regarding their adolescents’ PA during and after the intervention period.

**M2:** My kid used to go by scooter but this fall he used it once; the rest of the time he has taken the bike. You can see that it has had an effect. I asked him why he does not drive to school. He said he preferred to get a little exercise in the morning.

In some cases, the increased PA level came sometime after the end of the intervention. The parents also mentioned that they had noticed that the increased PA had led to positive effects on their adolescents’ energy levels, their ability to concentrate and learn in school, enhanced self-confidence, and improved mood. According to the parents this was because PA enhanced the adolescents’ endurance, motivation, focus, and skillfulness at planning their time, while some activities taught the adolescents to function well in cooperation with others.

**F8:** She feels that school works better. I have not had to “crack the whip” as much as earlier this year. She takes much more responsibility concerning school. She says that she feels soggy if she is not physically inactive. She plans for exercise when she is meeting her friends.

The parents reported that even though the intervention targeted PA it had also produced positive effects on their adolescents’ diets; they were more aware of what constitutes a good diet and more inclined to choose healthy options and cut down on sugar. However, a few parents expressed that it was a balancing act for their adolescents to not do too much PA; in some cases parents had tried to persuade their adolescents to take a day off from PA.

As well as the effect among the adolescents, the parents experienced some effects for themselves. They reported that their adolescents’ participation in the intervention had led to an increase in their own PA. PA had become a topic of conversation at home and they felt motivated to be more active, both with their adolescents and on their own. The intervention's impact on their own PA was unexpected, but the parents noted it with pleasure and reported that their adolescents also had made remarks concerning their enhanced PA levels.

**F9:** I have started to be more physically active this fall. I go to the gym, so it has rubbed off on me.

## Discussion of methods

We chose a qualitative approach, through individual interviews and content analysis, to describe parents’ experiences of being a part of their adolescents’ empowerment-inspired PA intervention. According to Öhman (2005), credibility can be increased by triangulation; the research problem is viewed from various angles by a research team with different professional backgrounds. To make use of this technique, the authors took an active part in the analysis of the data. We also used peer debriefing: the preliminary results were discussed with colleagues with experience of working with qualitative methods (Öhman, 2005). To enhance the credibility, we moved between the theme, subtheme, and the interview text to ensure that we included all data in the study and to ensure that our interpretations are reasonable. To help the readers consider our interpretations and to further give examples of different aspects, we included quotations in the results. Participants included mothers, fathers, boys, and girls, all with varying PA levels. This variety ensured that there was a good possibility that light would be shed on different aspects of the topic under investigation, thereby strengthening the credibility of the study (Graneheim & Lundman, 2004). We used an interview guide comprised of a few main questions with follow-up questions and kept an open dialogue within the research team, which according to Graneheim and Lundman (2004) strengthens the dependability of the data collection. Both positive and negative experiences were revealed, which suggests openness from the participants. Although these results are based on a small sample (only 10 out of 27 parents agreed to participate), the rich information in the interviews displayed a variety of experiences and was judged as sufficient to answer the aim of this study. In order to facilitate transferability, the research process, the participants, and the results have been described in detail. Authors can offer suggestions concerning the transferability; however, as stated by Graneheim and Lundman (2004), the transferability of the results to other contexts has to be considered by the reader.

## Discussion of results

Increasing adolescents’ PA can be difficult, especially when adolescents have many appealing competing priorities and different levels of interest in PA. The results of this study showed that the parents experienced the intervention as successful and that parents are an important part of increasing the PA of their adolescents. Therefore, parental involvement in the PA of adolescents should not be ignored. This finding is in line with earlier research (Edwardson & Gorely, 2010; Van Lippevelde et al., 2012), and this study outlines one way of including parents in an intervention to increase adolescents’ PA. In addition, we report an unexpected benefit of promoting the parents’ own PA.

Swedish adolescents of today do not reach an adequate PA level (Folkhälsomyndigheten, 2014). The findings of the present study indicate that there is a discrepancy between the actual PA performed by the adolescents and the level the parents think they ought to reach. The parents stated that they were more physically active when they were young, and this statement might be supported by the fact that there was a decline in cardiovascular fitness in Swedish 16-year-olds between 1987 and 2007 (Ekblom, Ekblom Bak, & Ekblom, 2011). We found that parents emphasized how their adolescents face greater challenges in prioritizing PA compared to their own childhoods and that this is due to societal changes. According to the results, adolescents spend a considerable amount of time with sedentary activities such as computers and mobile phones. This is in line with findings from other studies (Tercyak, Abraham, Graham, Wilson, & Walker, 2009) and consistent with an earlier focus group study with the students whose parents participated in this current study (Lindqvist et al., 2012). The results show that limited time acts as a barrier to prioritizing PA, which is in line with other studies (Patino-Fernandez et al., 2013; Thompson et al., 2010). Furthermore, a study of the connection between screen time and PA showed that every additional hour committed to PA was associated with 32 min less screen time and that this relationship was more pronounced in obese adolescents, who averaged 56 min less screen time (Olds, Ferrar, Gomersall, Maher, & Walters, 2012). Therefore, we argue that adolescents of today need support in choosing to participate in PA and that parents should be involved in providing this support.

The parents felt that it was mainly their responsibility to support their adolescents’ PA. This finding is in contrast with the findings of Patino-Fernandez et al. (2013), who reported that parents felt the school should provide children with participation in PA, while the school staff stated that it was the parents’ primary responsibility. However, the study concluded that there is a need for comprehensive school-based interventions emphasizing parent and school staff collaboration, and this is in line with our findings. The parents felt that school was the right context for a PA intervention, because it reached every adolescent regardless of their other life circumstances. This is consistent with the conclusions of other studies that state that most school settings provide the opportunity, equipment, facilities, and staffing needed to effectively promote PA (Carson et al., 2014; Naylor & McKay, 2009). This is also supported by social cognitive theory, which extends the conception of individuals to the collective, as people do not operate as isolated individuals, but work together to improve their health (Bandura, 2004). Even though health-promoting interventions should be implemented in school, they should not be performed solely by school staff. School-based health-promoting interventions could benefit from integrating support from home, the community, and society at large (Bandura, 2004). We agree with Bandura that school is the appropriate place to implement a health-promoting intervention that aims to reach children and adolescents. Still, this matter is not without complications. Society tends to focus on areas that are evaluated, and schools are not graded on health promotion (Bandura, 2004). Furthermore, promoting health might appear to be an added burden when the primary focus of schools is to meet academic standards; PA is sometimes seen as a competitor to academic studies, because the time devoted to PA could instead be devoted to academic work (Ickovics et al., 2014).

The parents in this study noted how their own PA behavior and actions as a positive role model influenced their adolescents’ PA. The connection between the parents’ level of PA and their children's level of PA is also mentioned by Khanolkar, Byberg, and Koupil (2012), who stated that children can benefit if their parents adopt a healthy lifestyle. In addition, encouragement and help with practical issues and logistics also impacted their adolescents’ PA, which is consistent with the review by Edwardson and Gorely (2010) and other studies (Cheng, Mendonça, & De Farias Júnior, 2014; Khanolkar et al., 2012). Furthermore, the support provided by parents included limit setting on sedentary behavior, which they stated to be important not only with smaller children but also with adolescents, which is in agreement with other studies (Alia, Wilson, St George, Schneider, & Kitzman-Ulrich, 2013; Ramirez et al., 2011). Moreover, the parents emphasized the importance of creating a healthy lifestyle at a young age, since according to them it is difficult to change behavior later in life. Two reviews on tracking of PA from childhood to adulthood support this statement and conclude that enhancement of PA in children is of great importance for the promotion of public health (Craigie, Lake, Kelly, Adamson, & Mathers, 2011; Telama et al., 2014), a conclusion this study supports.

Moreover, we argue that the support of PA needs to be coordinated to fit within the adolescents’ reality as “digital natives.” Prensky (2001) reported that adolescents think and process information fundamentally differently from their predecessors as a result of being surrounded by new technology. These digital natives are compared with the older generation of “digital immigrants,” who are learning and adopting new technology (Prensky, 2001). An empowerment element in an intervention can contribute to the extent to which the intervention fits the end users’ priorities and values (Fraser & Galinsky, 2010). According to the parents, it was an advantage that the intervention was empowerment-inspired and built on the adolescents’ own ideas; they believed that this design might enhance the possibility of sustainability. It is possible to draw parallels between this study and the findings of Puolakka and colleagues, who concluded that engaging students not only made them more confident but also contributed to their health (Puolakka, Haapasalo-Pesu, Konu, Åstedt-Kurki, & Paavilainen, 2014). As a result of their project, the physical condition and social relationships of the students improved, and future interventions were planned (Puolakka et al., 2014). Furthermore, the present study showed that the parents felt empowered after reading the parental information brochure made by their adolescents, which increased self-efficacy and confirmed that some of their previous attitudes and actions had been correct. This impact is important, because a feeling of helplessness and lack of control may disempower parents and ultimately lead to behaviors that are unsupportive of their adolescents (Patino-Fernandez et al., 2013). Interestingly, the parents who stated that their adolescents do not always have the ability to know what is best for them or have the capacity to understand the long-term consequences of their actions were impressed by their adolescents’ knowledge and their ability to find the right details to create the parental information. Taken together, the results of this study suggests that empowerment and the forming of partnerships between significant adults and adolescents is a promising avenue for developing PA interventions, which echoes social cognitive theory (Bandura, 2004). However, further research will be needed to clarify this point.

The parents found that the intervention had a positive effect upon their adolescents’ PA. This observation is consistent with the findings from our previous quantitative study, which showed an increase in PA of 4.9 min per day (Lindqvist, Mikaelsson, et al., 2014). Furthermore, this is in line with the results from our previous interview study with adolescents (Lindqvist, Kostenius, & Gard, 2014). The interviews were performed 2 and 8 weeks after the intervention had ended for adolescents and their parents, respectively, suggesting the possibility of a lasting effect for the intervention. Moreover, the parents reported that, although the intervention targeted PA, it had also had a positive effect upon their adolescents’ diet. Clustering of health-related behaviors is known to result in synergistic effects, when a change in one behavior affects the prevalence of another, although the relationship is complex and requires further research (Busch, Van Stel, Schrijvers, & De Leeuw, 2013; Leech et al., 2014). Knowledge of health behavioral clustering can be used to design more effective school-based interventions using transfer-oriented learning, thus targeting multiple behaviors simultaneously (Busch et al., 2013).

The effect of the intervention was also transmitted from the adolescents to the parents, because the parents experienced that the intervention had led to an increase in their own PA. To our knowledge, this phenomenon has not been previously reported. PA became a topic of conversation at home in our study. This factor echoes the key messages of Thompson et al. (2010), who stated that participating in a PA intervention that includes a family component can enhance parent-child communication and social interaction among family members.

## Conclusions

In this study, parents observed that the intervention positively affected their adolescents’ PA, an effect that continued after the end of the intervention and additionally benefitted the parents’ own PA. The findings indicate that the parents were important as role models, providing encouragement and tangible support. Therefore, we suggest that interventions aimed at promoting PA among adolescents should include actions to stimulate participation of parents or other significant adults. Preferably, the intervention should be school-based and have an empowerment approach to ensure a solution implemented to fit within the adolescents’ reality.

## References

[CIT0001] Alia K. A, Wilson D. K, St George S. M, Schneider E, Kitzman-Ulrich H (2013). Effects of parenting style and parent-related weight and diet on adolescent weight status. Journal of Pediatric Psychology.

[CIT0002] Bandura A (2004). Health promotion by social cognitive means. Health Education and Behavior.

[CIT0003] Basch C. E (2011). Healthier students are better learners: High-quality, strategically planned, and effectively coordinated school health programs must be a fundamental mission of schools to help close the achievement gap. Journal of School Health.

[CIT0004] Beets M, Cardinal B, Alderman B (2010). Parental social support and the physical activity-related behaviors of youth: A review. Health Education & Behavior.

[CIT0005] Birch L. L, Ventura A. K (2009). Preventing childhood obesity: What works?. International Journal of Obesity.

[CIT0006] Busch V, Van Stel H, Schrijvers A, De Leeuw J (2013). Clustering of health-related behaviors, health outcomes and demographics in Dutch adolescents: A cross-sectional study. BMC Public Health.

[CIT0007] Carson R. L, Castelli D. M, Beighle A, Erwin H (2014). School-based physical activity promotion: A conceptual framework for research and practice. Childhood Obesity.

[CIT0008] Cheng L, Mendonça G, De Farias Júnior J (2014). Physical activity in adolescents: Analysis of the social influence of parents and friends. Jornal de pediatria.

[CIT0009] Craigie A. M, Lake A. A, Kelly S. A, Adamson A. J, Mathers J. C (2011). Tracking of obesity-related behaviours from childhood to adulthood: A systematic review. Maturitas.

[CIT0010] Donnelly J. E, Lambourne K (2011). Classroom-based physical activity, cognition, and academic achievement. Preventive Medicine.

[CIT0011] Edwardson C. L, Gorely T (2010). Parental influences on different types and intensities of physical activity in youth: A systematic review. Psychology of Sport and Exercise.

[CIT0012] Ekblom Ö. B, Ekblom Bak E. A. M, Ekblom B. T (2011). Cross-sectional trends in cardiovascular fitness in Swedish 16-year-olds between 1987 and 2007. Acta Paediatrica.

[CIT0013] Elo S, Kyngäs H (2008). The qualitative content analysis process. Journal of Advanced Nursing.

[CIT0014] Folkhälsomyndigheten (2014). Skolbarns hälsovanor i sverige 2013/14 [Health Behaviour in School-aged Children (HBSC), results from Sweden of the 2013/14 WHO study]. http://www.folkhalsomyndigheten.se/publicerat-material/publikationer/Skolbarns-halsovanor-i-Sverige-201314/.

[CIT0015] Fraser M. W, Galinsky M. J (2010). Steps in intervention research: Designing and developing social programs. Research on Social Work Practice.

[CIT0016] Graneheim U. H, Lundman B (2004). Qualitative content analysis in nursing research: Concepts, procedures and measures to achieve trustworthiness. Nurse Education Today.

[CIT0038] Ickovics J. R, Carroll-Scott A, Peters S. M, Schwartz M, Gilstad-Hayden K, McCaslin C (2014). Health and academic achievement: Cumulative effects of health assets on standardized test scores among urban youth in the united states. Journal of School Health.

[CIT0017] Janssen I, LeBlanc A. G (2010). Systematic review of the health benefits of physical activity and fitness in school-aged children and youth. International Journal of Behavioral Nutrition and Physical Activity.

[CIT0018] Khanolkar A. R, Byberg L, Koupil I (2012). Parental influences on cardiovascular risk factors in Swedish children aged 5–14 years. The European Journal of Public Health.

[CIT0019] Kvale S, Brinkmann S (2009). Den kvalitativa forskningsintervjun [The qualitative research interview].

[CIT0020] Leech R. M, McNaughton S. A, Timperio A (2014). The clustering of diet, physical activity and sedentary behavior in children and adolescents: A review. International Journal of Behavioral Nutrition and Physical Activity.

[CIT0021] Lindqvist A. K, Kostenius C, Gard G (2012). “Peers, parents and phones”—Swedish adolescents and health promotion. International Journal of Qualitative Studies on Health and Well-Being.

[CIT0022] Lindqvist A. K, Kostenius C, Gard G (2014). Fun, feasible and functioning: Students’ experiences of a physical activity intervention. European Journal of Physiotherapy.

[CIT0023] Lindqvist A. K, Mikaelsson K, Westerberg M, Gard G, Kostenius C (2014). Moving from idea to action: Promoting physical activity by empowering adolescents. Health Promotion Practice.

[CIT0024] Mengshoel A. M (2012). Mixed methods research—So far easier said than done?. Manual Therapy.

[CIT0025] Naylor P. J, McKay H. A (2009). Prevention in the first place: Schools a setting for action on physical inactivity. British Journal of Sports Medicine.

[CIT0026] Öhman A (2005). Qualitative methodology for rehabilitation research. Journal of Rehabilitation Medicine.

[CIT0027] Olds T, Ferrar K. E, Gomersall S. R, Maher C, Walters J. L (2012). The elasticity of time: Associations between physical activity and use of time in adolescents. Health Education and Behavior.

[CIT0028] Patino-Fernandez A. M, Hernandez J, Villa M, Delamater A (2013). School-based health promotion intervention: Parent and school staff perspectives. Journal of School Health.

[CIT0029] Prensky M (2001). Digital natives, digital immigrants, part 1. On the Horizon.

[CIT0030] Puolakka K, Haapasalo-Pesu K, Konu A, Åstedt-Kurki P, Paavilainen E (2014). Mental health promotion in a school community by using the results from the well-being profile. Health Promotion Practice.

[CIT0031] Ramirez E. R, Norman G. J, Rosenberg D. E, Kerr J, Saelens B. E, Durant N (2011). Adolescent screen time and rules to limit screen time in the home. Journal of Adolescent Health.

[CIT0039] Rauscher L, Greenfield B. H (2009). Advancements in contemporary physical therapy research: Use of mixed methods designs. Physical Therapy.

[CIT0032] Telama R, Yang X, Leskinen E, Kankaanpää A, Hirvensalo M, Tammelin T (2014). Tracking of physical activity from early childhood through youth into adulthood. Medicine and Science in Sports and Exercise.

[CIT0033] Tercyak K. P, Abraham A. A, Graham A. L, Wilson L. D, Walker L. R (2009). Association of multiple behavioral risk factors with adolescents’ willingness to engage in eHealth promotion. Journal of Pediatric Psychology.

[CIT0034] Thompson J, Jago R, Brockman R, Cartwright K, Page A, Fox K (2010). Physically active families—De-bunking the myth? A qualitative study of family participation in physical activity. Child Care Health and Development.

[CIT0035] Van Lippevelde W, Verloigne M, De Bourdeaudhuij I, Brug J, Bjelland M, Lien N (2012). Does parental involvement make a difference in school-based nutrition and physical activity interventions? A systematic review of randomized controlled trials. International Journal of Public Health.

[CIT0036] World Health Organization (2010). Global recommendations on physical activity for health.

[CIT0037] Wright M. S, Wilson D. K, Griffin S, Evans A (2010). A qualitative study of parental modeling and social support for physical activity in underserved adolescents. Health Education Research.

